# Endothelial GABBR2 Regulates Post-ischemic Angiogenesis by Inhibiting the Glycolysis Pathway

**DOI:** 10.3389/fcvm.2021.696578

**Published:** 2021-08-04

**Authors:** Hongze Zhang, Hong Zhou, Jianying Yuan, Yong Nan, Jingquan Liu

**Affiliations:** ^1^Department of Emergency Medicine and Critical Care, Shanghai Songjiang District Central Hospital, Songjiang Hospital Affiliated to Shanghai Jiaotong University School of Medicine (Preparatory Stage), Shanghai, China; ^2^Department of Intensive Care Unit, Zhejiang Provincial People's Hospital, Hangzhou, China; ^3^Department of Intensive Care Unit, People's Hospital of Hangzhou Medicine College, Hangzhou, China

**Keywords:** GABBR2, angiogenesis, glycolysis, endothelial (dys)function, ischemic injury

## Abstract

**Purpose:** Angiogenesis post-ischemia plays an essential role in preventing ischemic damage to tissue by improving the blood recovery. Determining the regulatory mechanism of ischemic angiogenesis, therefore, could provide effective therapeutics for ischemic injury.

**Materials and Methods:** The RNA sequencing (RNA-seq) database was used to predict the association of gamma-aminobutyric acid type B receptor subunit 2 (GABBR2) with endothelial-specific expression. The role of GABBR2 in angiogenesis was verified *in vitro* by downregulating GABBR2 in human umbilical vein endothelial cells (HUVECs) with lentiviral vectors. Besides, the *in vivo* effect of GABBR2 on the blood recovery of an ischemic hindlimb was demonstrated by establishing a hindlimb ischemia model in normal and GABBR2 adenoviral vector-infected mice. Then, the mobilization of endothelial progenitor cells (EPCs) in peripheral blood post-ischemia was determined by flow cytometry. Finally, the XF analyzer and Western blot were used to determine the effect of GABBR2 on endothelial metabolism.

**Results:** The RNA-seq results indicated a strong association between GABBR2 and endothelial revascularization, and the upregulation of GABBR2 was detected in both hypoxia-treated HUVECs and ischemic mouse hindlimb. Hypoxia treatment for 6 h increased the proliferation, migration, and tube formation of HUVECs, which were inhibited by GABBR2 knockdown. Additionally, GABBR2 downregulation significantly decreased the blood flow recovery of mouse ischemic hindlimb. The expressions of the EPC markers CD34^+^ and CD133^+^ significantly decreased in the peripheral blood in hindlimb post-ischemia. Mechanically, glycolysis-dominated metabolism of HUVECs was compromised by GABBR2 knockdown. Evidences of the decreased expressions of HKII, PFKFB3, and PKM1 also supported the compromised glycolysis induced by GABBR2 downregulation.

**Conclusion:** Our study demonstrated that GABBR2 regulated angiogenesis post-ischemia by inhibiting the glycolysis pathway.

## Introduction

The rapid and timely reestablishment of blood vessels in response to injury or in pathological conditions ensures the efficient supply of oxygen and nutrients, which are essential for the protection of tissues from ischemic injury. Peripheral arterial diseases (PADs) are associated with dysfunctional collateral circulation and are linked to a high risk of cardiovascular events, specifically stroke and myocardial infarction (1). Illustrating the mechanism of endothelial cell (EC) angiogenesis could provide therapeutic targets in ameliorating the development of cardiovascular events.

Current studies majorly focus on the growth factors, receptors, signaling molecules, and others involved in the process of angiogenesis, but no significant efficacy or specificity was revealed so far. Recent findings have demonstrated that pathological angiogenesis and EC dysfunction are accompanied by EC-specific metabolic alterations. Targeting EC metabolism is therefore emerging as a novel therapeutic strategy (2–4). The switch of ECs from quiescence to growth metabolically demands that 85% ATP is produced glycolytically (3, 5, 6). Even though glucose oxidative metabolism produces 34 molecules of ATP, ECs still preferentially utilize glycolysis instead of oxidative metabolism. As glycolytic regulators, 6-phosphofructo-2-kinase/fructose-2,6-bisphosphatase 3 (PFKFB3) and hexokinase2 (HK2) are rate-limiting enzymes that regulate EC angiogenesis by modulating glycolytic metabolism. Silencing of the glycolytic regulators PFKFB3 and HK2 inhibited the atrial development and branching of mouse skin and the proliferation of filopodia in the retinal vasculature (5, 7). The reasons for glycolysis-dependent metabolism might lie in ECs bathing in oxygen needing anaerobic metabolism to protect themselves from oxidative stress. The stored oxygen is diffused to perivascular cells. In addition, glycolysis yields fewer but faster ATP, which is necessary for the angiogenesis of ECs in hypoxic tissues.

Gamma-aminobutyric acid (GABA) receptors are heterodimeric G-protein-coupled receptors comprising the GABAB1 and GABAB2 subunits (GABBR2) (8). Gamma-aminobutyric acid receptors are located mainly in the central nervous system (CNS) and retina. Additionally, GABA receptors have also been detected in peripheral tissues and are shown to modulate intracellular calcium concentration (9, 10). These receptors can inhibit the release of many neurotransmitters, such as dopamine, serotonin, and acetylcholine, *via* G-protein-dependent inhibition of neuronal voltage-gated Ca^2+^ channels. Functional GABA receptors are implicated in various disorders, including cognitive impairments, nociception, anxiety, depression, and epilepsy (11, 12). Gamma-aminobutyric acid is a neurotrophic factor that plays an important role in embryonic development of the nervous system by promoting the proliferation, migration, and differentiation of embryonic neural cells and neural crest cells (13, 14). Additionally, GABA receptors regulate intracellular signaling by inhibiting adenylyl cyclase activity (15). Here, we found a potential of GABBR2 in regulating EC function. Then, the effect of GABBR2 on angiogenesis capacity was established by downregulating GABBR2 *in vivo* and *in vitro*. Our present results illustrated that GABBR2 knockdown inhibited the angiogenesis process post-ischemia by regulation of the glycolysis-dominant metabolism.

## Materials and Methods

### Animals

Adult C57BL/6J male mice (weighing 20–22 g), obtained from Shanghai Research Center for Model Organisms, were used in this study. Care and all animal experiments were performed in accordance with institutional guidelines. Mice were housed at room temperature under a 12:12 light/dark cycle with free access to water and standard laboratory mouse chow.

### Murine Hindlimb Ischemia Model

The fur in the hindlimb of adult C57BL/6J male mice were removed after anesthetized by intraperitoneal injection of pentobarbital sodium (40 mg/kg body weight). Then, the skin of the left hindlimb was dissected and the femoral artery exposed. Ischemia of the hindlimb was induced by ligation and excision of the femoral artery at middle and two terminals.

### Perfusion Recovery Detection

Blood flow recovery in the ischemic hindlimb was analyzed by laser Doppler perfusion imaging (PeriScan PIM 3 System, Perimed, Stockholm, Sweden) on days 1, 3, 5, 7, and 10. In detail, the mice were anesthetized by intraperitoneal injection of pentobarbital sodium and placed on 37°C heating plate, and then the hindquarters were imaged by laser Doppler imaging. Blood flow recovery was calculated as the perfusion ratio of ischemic limbs vs. non-ischemic limbs.

### Cell Culture

Human umbilical vein endothelial cells (HUVECs) were obtained from Cell Bank of Chinese Academy of Science (Shanghai, China) and cultured in Dulbecco's modified Eagle's medium (DMEM) with 10% fetal bovine serum (FBS) at 37°C in a humidified incubator with 21% O_2_ and 5% CO_2_. Hypoxia treatment was performed by culturing the cells in an incubator with 1% O_2_ and 5% CO_2_. For GABBR2 knockdown in HUVECs, the lentiviral vectors were infected for at least 12 h, then the medium was replaced with a virus-free medium.

### Tubule Formation Assay and CCK-8 Assay

The angiogenic effect of HUVECs was quantified by tubule formation assay in Matrigel (cat no. 354234, Corning, Corning, NY, USA). The Matrigel solution was thawed overnight at 4°C, and then 200 μl thawed Matrigel was transferred and plated onto a 24-well plate per well. After incubating for 30 min at 37°C, 10^4^ HUVECs were resuspended with endothelial cell basal medium (ECM-2, Lonza, Basel, Switzerland) and then seeded onto the Matrigel. After 6 h, the tube formation status was imaged by a microscope (Nikon TE2000, Nikon, Tokyo, Japan). For the CCK-8 assay, Cell Counting Kit-8 (Weiao Co., Shanghai, China) was used for the assessment of cellular proliferation.

### Flow Cytometry Assay

To assess the endothelial progenitor cell (EPC) levels mobilized post-ischemic injury, peripheral blood samples were harvested at day 7 post-hindlimb ischemia in mice. Erythrocyte lysis buffer (BD Biosciences, Franklin Lakes, NJ, USA) was used to lyse red blood cells in a shaker at room temperature for 10 min. After fixing with 4% paraformaldehyde, the cells were stained with the antibodies CD34-FITC (11-0349-42, eBioscience, San Diego, CA, USA) and CD133-PE (12-1331-82, eBioscience). Stained cells were then analyzed on a FACScan flow cytometer (Becton Dickinson, Franklin Lakes, NJ, USA).

### Adenoviral Vector Construction and Transfection

The AAV9–GABBR2–shRNA–zsgreen vector was used in this study for the downregulation of GABBR2 expression in mouse hindlimb. Adenovirus was injected intramuscularly according to the manufacturer's instruction. Of Ad.GABBR2 or Ad.Null, 5 ×10^9^ plaque-forming units (PFU) in 100 μl of phosphate-buffered saline (PBS) was injected into the gastrocnemius muscle of mice 4 weeks before surgery, respectively.

### Western Blot

Cultured cells and tissue from mouse hindlimb were lysed on ice with RIPA buffer (Sigma-Aldrich, St. Louis, MO, USA) containing protease inhibitor cocktail (ThermoFisher Scientific, Waltham, MA, USA) and PhosSTOP (ThermoFisher Scientific) for at least 30 min. After centrifugation at 12,000 rpm for 10 min at 4°C, supernatant fractions were collected and analyzed by sodium dodecyl sulfate polyacrylamide gel electrophoresis (SDS-PAGE) and immunoblotting. The following antibodies were obtained from Cell Signaling (Danvers, MA, USA): GABA(B)R2 antibody (#3839, 1:1,000), Hexokinase II (HKII) antibody (#2867, 1:1,000), and pyruvate kinase muscle 1 (PKM1) antibody (#7067, 1:1,000). Vascular endothelial growth factor (VEGF) antibody (#65373, 1:1,000), anti-PFKFB3 (ab181861, 1/1,000–1/10,000), and anti-CD31 (ab76533, 1/5,000–1/20,000) were obtained from Abcam (MitoSciences-Abcam, Eugene, OR, USA). Horseradish peroxidase (HRP)-conjugated monoclonal mouse anti-beta actin (KC-5A08, 1:5,000) was obtained from KangChen Bio-tech Co. (Shanghai, China).

### Bioenergetics Assay of ECAR Changes

The extracellular acidification rates (ECARs) of the HUVECs in different groups were analyzed using an XFe96 extracellular flux analyzer (Seahorse Bioscience, Billerica, MA, USA). The optimal seeding density of HUVECs ranges from 10,000 to 60,000 cells per well and gives confluency of approx. 95% overnight in a 96-well seahorse culture plate. ECAR was determined by adding glucose (10 mM), oligomycin A (1 μM), and 2-deoxy-d-glucose (1 M).

### Immunofluorescent Staining

The quadriceps femoris muscles of wild-type (WT) and GABBR2 knockdown (KD) ischemic limbs were harvested and embedded with OCT. Then, the embedded tissues were sectioned at 8 μm. After fixation and blocking, the sections were incubated with goat anti-mouse CD31 antibody (1:200, NOVUS Biologicals, Littleton, CO, USA). Alexa Fluor 549 donkey anti-goat secondary antibody (1:150, ThermoFisher Scientific) was used for fluorescence imaging.

### EdU Staining Assay

The effect of GABBR2 on the proliferative abilities of HUVECs was detected by using the BeyoClick EdU Cell Proliferation Kit with Alexa Fluor 488 (Beyotime, Jiangsu, China). In detail, 100 μM 5-ethynyl-2′-deoxyuridine (EdU) solution was added to the medium and incubated with HUVECs for 2 h. The divided cells stained with green represented EdU-positive cells.

### Statistical Analyses

Statistical analyses were performed using one-way ANOVA with Tukey's correction using GraphPad 8.0 software. Statistical results were shown as the mean ± standard error of the mean (SEM). Every experiment was repeated at least three times.

## Results

### GABBR2 Was Closely Related to Endothelial Angiogenesis and Upregulated by Hypoxia Treatment

**Figure 1:**
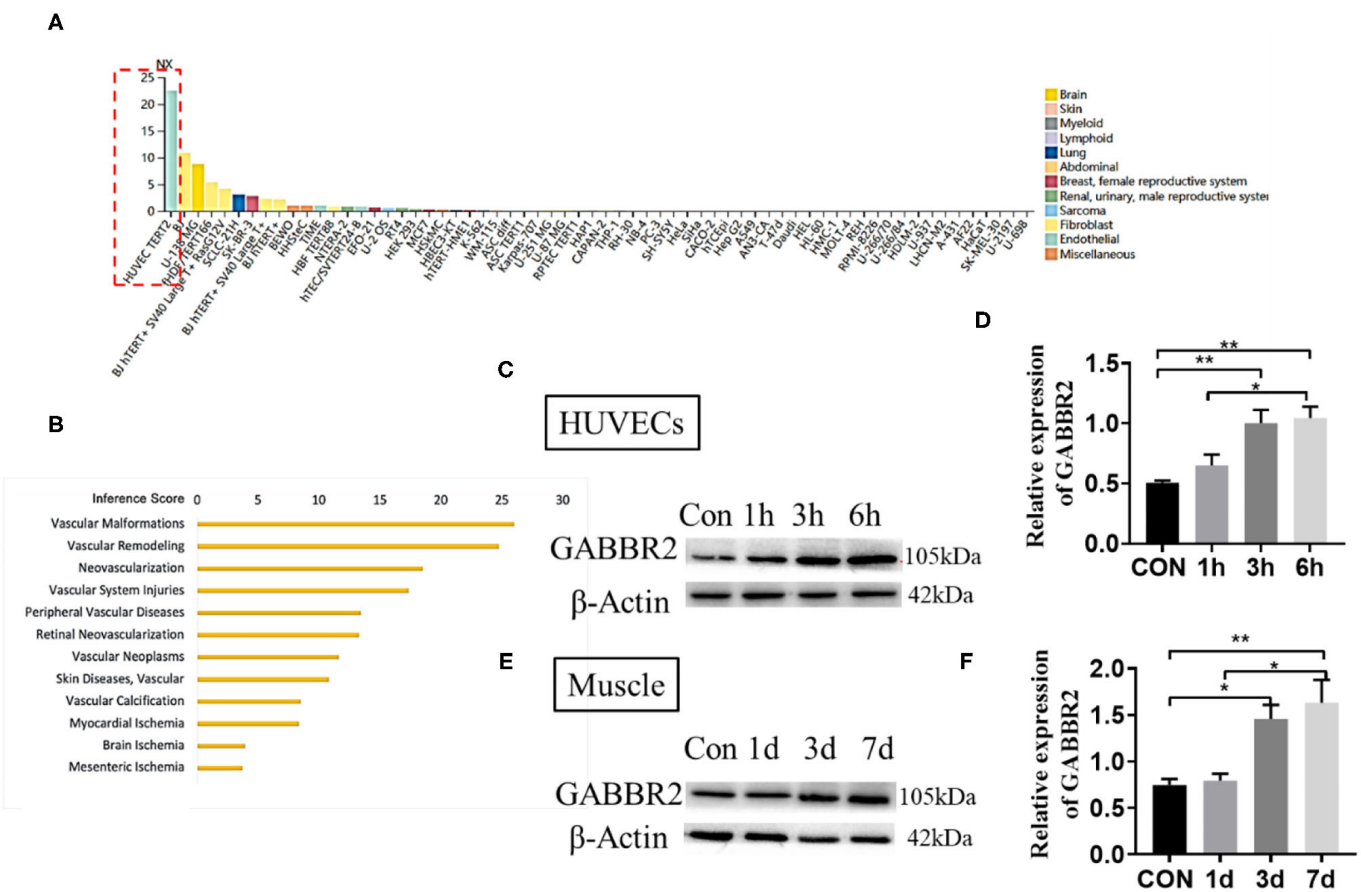
Gamma-aminobutyric acid type B receptor subunit 2 (GABBR2) expression increased in hypoxia-treated endothelial cells *in vitro* and mouse ischemic hindlimb *in vivo*. **(A)** Prediction of an endothelial-specific expression of GABBR2 by RNA-seq data. **(B)** Re-speculation of the role of GABBR2 in angiogenesis. **(C,D)** Expression changes of GABBR2 in human umbilical vein endothelial cells (HUVECs) after hypoxia treatment for 1, 3, and 6 h. **(E,F)** Expression changes of GABBR2 in the muscle of mouse ischemic hindlimb on days 1, 3, and 7. *p <0.05; **p <0.01.

The RNA sequencing (RNA-seq) data from the Human Protein Atlas (https://www.proteinatlas.org/) predicted an endothelial-specific expression of GABBR2 (Figure 1A). We therefore speculated that GABBR2 might play an important role in the regulation of angiogenesis. To obtain the genes involved in angiogenesis, we resorted to the publicly available ANGIOGENES database of RNA-seq (http://angiogenes.uni-frankfurt.de), identifying 14,153 genes whose transcription was dynamically modulated during VEGF stimulation (12 h) of HUVECs. Gamma-aminobutyric acid type B receptor subunit 2 was one of the genes closely related to physical or pathological angiogenesis (Figure 1B). Hypoxia was a driver that regulates angiogenesis and vascular remodeling (16). To explore the effect of hypoxia on the regulation of GABBR2, we cultured HUVECs under the condition of 1% oxygen for 0, 6, 12, and 24 h. The results in Figures 1C,D demonstrated a significantly enhanced expression of GABBR2 in HUVECs after hypoxia treatment. Furthermore, to determine the role of GABBR2 in mouse vascular ECs, we performed a hindlimb ischemia model and detected the expression of GABBR2 in the ischemic hindlimb muscle at different time points. The results also showed a markedly upregulated expression of GABBR2 in hindlimb post-ischemia (Figures 1E,F). The above results indicated that GABBR2 might play an important role in regulating the angiogenesis of ECs post-hypoxia or ischemia.

### GABBR2 Downregulation Regulated the Angiogenesis of HUVECs With or Without Hypoxia Treatment

**Figure 2:**
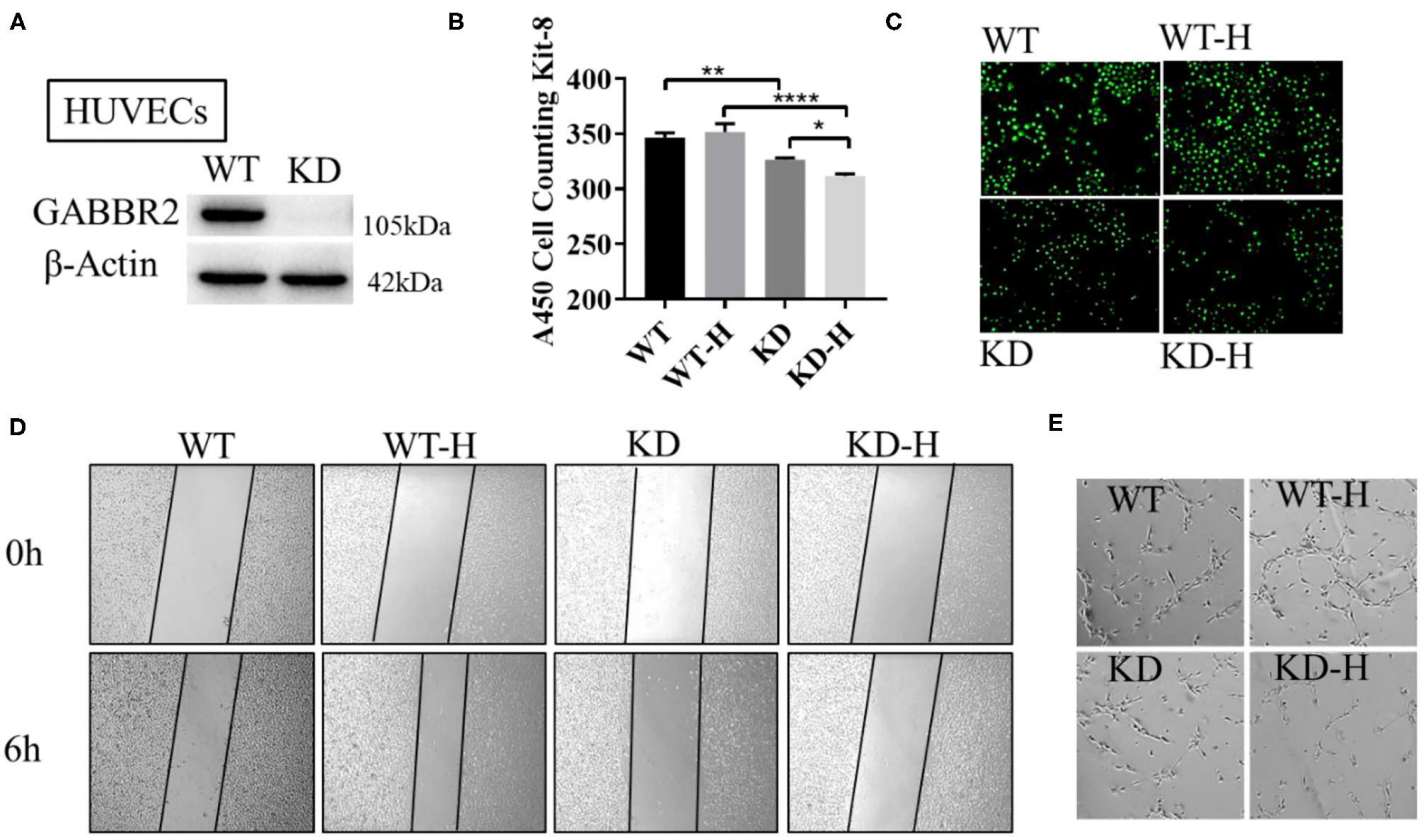
Effect of gamma-aminobutyric acid type B receptor subunit 2 (GABBR2) knockdown on the proliferation, migration, and angiogenesis of human umbilical vein endothelial cells (HUVECs). **(A)** Downregulation of GABBR2 in HUVECs by lentivirus transfection. **(B)** Cellular proliferation changes induced by 6 h of hypoxic treatment in normal and GABBR2-downregulated HUVECs. **(C)** EdU staining induced by 6 h of hypoxic treatment in normal and GABBR2-downregulated HUVECs. **(D)** Wound healing distance changes induced by 6 h of hypoxic treatment in normal and GABBR2-downregulated HUVECs. **(E)** Tube-like construction formation induced by 6 h of hypoxic treatment in normal and GABBR2 downregulated HUVECs. *p < 0.05; **p < 0.01; ****p < 0.0001.

Human umbilical vein endothelial cells were used to further demonstrate the role of GABBR2 in angiogenesis. We constructed the lentivirus-mediated GABBR2 downregulation in HUVECs and analyzed the proliferation and migration capacity. The results showed that about 90% of the GABBR2 expression was downregulated after lentivirus transfection (Figure 2A). The CCK-8 assay results showed that GABBR2 knockdown significantly inhibited the proliferation of HUVECs under normoxia, which could be further inhibited after 6 h of hypoxic treatment (Figure 2B). Besides, the EdU staining assay demonstrated that GABBR2 knockdown significantly decreased the ratio of EdU-positive cells under normoxia and hypoxia (Figure 2C). Then, the migration capacity was calculated as the average wound healing distance; promotion of endothelial migration was detected after hypoxia treatment, which could be eliminated by GABBR2 knockdown (Figure 2D). Since angiogenesis capacity could be evaluated by a tube-like construction formation in Matrigel, we then assessed the effect of GABBR2 knockdown on the formation of capillary-like tubes. As shown in Figure 2E, GABBR2 knockdown significantly inhibited the capillary-like tube formation in HUVECs.

### GABBR2 Knockdown Inhibited the Blood Flow Recovery in Ischemic Mouse Hindlimb

**Figure 3:**
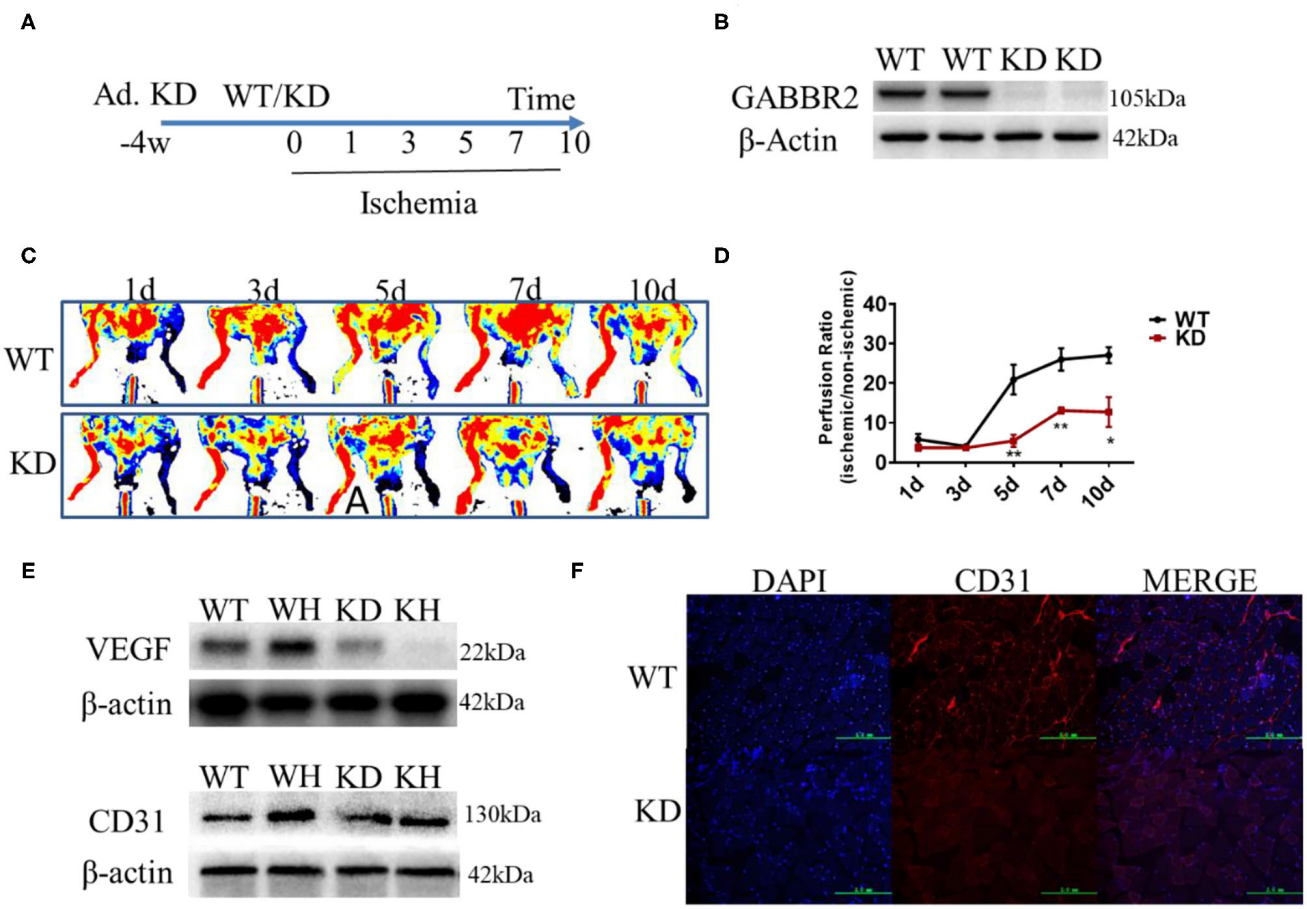
Effect of gamma-aminobutyric acid type B receptor subunit 2 (GABBR2) knockdown on blood recovery of the ischemic hindlimb in mice. **(A)** Protocol of adenovirus injection and the establishment of mouse hindlimb ischemia. **(B)** Knockdown efficiency of GABBR2 in mouse hindlimb. **(C)** Blood recovery of mouse hindlimb imaged by laser Doppler at different time points. **(D)** Statistical analysis of the blood perfusion ratio (ischemic/non-ischemic hindlimb). **(E)** Expression changes of VEGF and CD31 post-ischemia in wild-type (WT) and GABBR2-downregulated hindlimbs. **(F)** Immunofluorescent staining of CD31 in WT and GABBR2-downregulated ischemic hindlimbs. *Bar*, 200 μm. *p < 0.05; **p < 0.01.

Adenovirus encoding a constitutively inactive form of GABBR2 was injected in mouse hindlimb by multipoint for 4 weeks (Figure 3A). The Western blot results demonstrated that the expression of GABBR2 was downregulated by about 80% in the muscle of mouse hindlimb (Figure 3B). To determine the role of GABBR2 knockdown in angiogenesis and blood flow recovery, a hindlimb ischemia model was established in normal and GABBR2-downregulated mice. The blood flow recovery status on days 1, 3, 5, 7, and 10 was evaluated by laser Doppler imaging post-ischemia. The results indicated that GABBR2 downregulation significantly abrogated the blood flow recovery by 3.84, 1.98, and 2.13 times on days 5, 7, and 10, respectively (Figures 3C,D). To further prove the inhibitory effect of GABBR2 knockdown on the angiogenesis of mouse ischemic hindlimb, we determined the expression of VEGF and platelet endothelial cell adhesion molecule-1 (PECAM-1/CD31) in WT and GABBR2-downregulated hindlimbs. The results indicated that the upregulation of VEGF and CD31 induced by ischemia was inhibited by GABBR2 knockdown (Figure 3E). Immunofluorescent staining of CD31 in WT and GABBR2-downregulated hindlimbs showed consistent results in that GABBR2 knockdown decreased CD31 expression in ischemic hindlimb (Figure 3F). Overall, these evidences demonstrated that GABBR2 is an important regulator in promoting angiogenesis post-ischemia.

### GABBR2 Knockdown Decreased the Mobilization of EPCs in Peripheral Blood Post-ischemia

**Figure 4:**
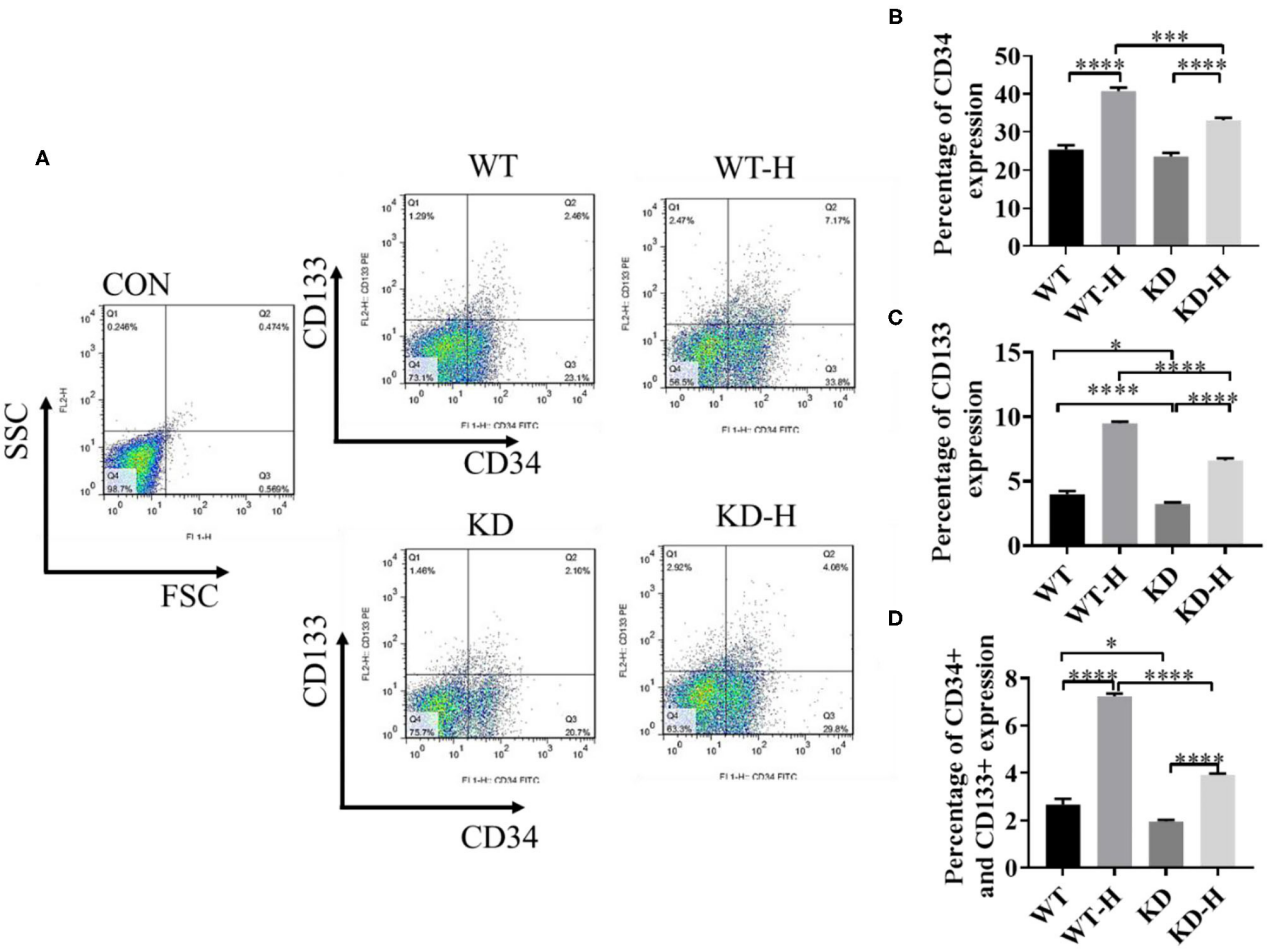
Effect of gamma-aminobutyric acid type B receptor subunit 2 (GABBR2) knockdown on endothelial progenitor cell (EPC) mobilization in peripheral blood in hindlimb post-ischemia. **(A)** Representative images of CD34^+^/CD133^+^ cells in the peripheral blood of mice analyzed by flow cytometry. *WT*, normal mice; *KD*, GABBR2-downregulated mice; *WT-H*, wild-type (WT) mice with hindlimb ischemia; *KD-H*, GABBR2 knockdown (KD) mice with hindlimb ischemia. **(B)** Ratio of CD34^+^ cells in the peripheral blood of normal and ischemic mice. **(C)** Ratio of CD133^+^ cells in the peripheral blood of normal and ischemic mice. **(D)** Ratio of CD34^+^ and CD133^+^ cells in the peripheral blood of normal and ischemic mice. *p < 0.05; ***p < 0.001; ****p < 0.0001.

CD34 and CD133 are the most commonly used EPC markers expressed in early and mature vascular ECs that play an important role in EPC proliferation and differentiation, therefore serving as a powerful pan-endothelial marker (17, 18). To determine the mobilization of EPCs, we collected peripheral blood from normal and ischemic mice with or without GABBR2–shRNA transfection. Then, we analyzed CD34- or CD133-positive cells and CD34/CD133 double-positive cells by using flow cytometry. The data showed that ischemia increased the ratio of CD34 and CD133 double-positive cells in peripheral blood from 2.45 to 7.17%, which was significantly blunted by GABBR2 knockdown (Figures 4A–D). These results implied that ischemia-induced mobilization of EPCs in peripheral blood was markedly inhibited by GABBR2 downregulation.

### GABBR2 Knockdown Inhibited the Angiogenesis of HUVECs by Regulating the Glycolysis Pathway

**Figure 5:**
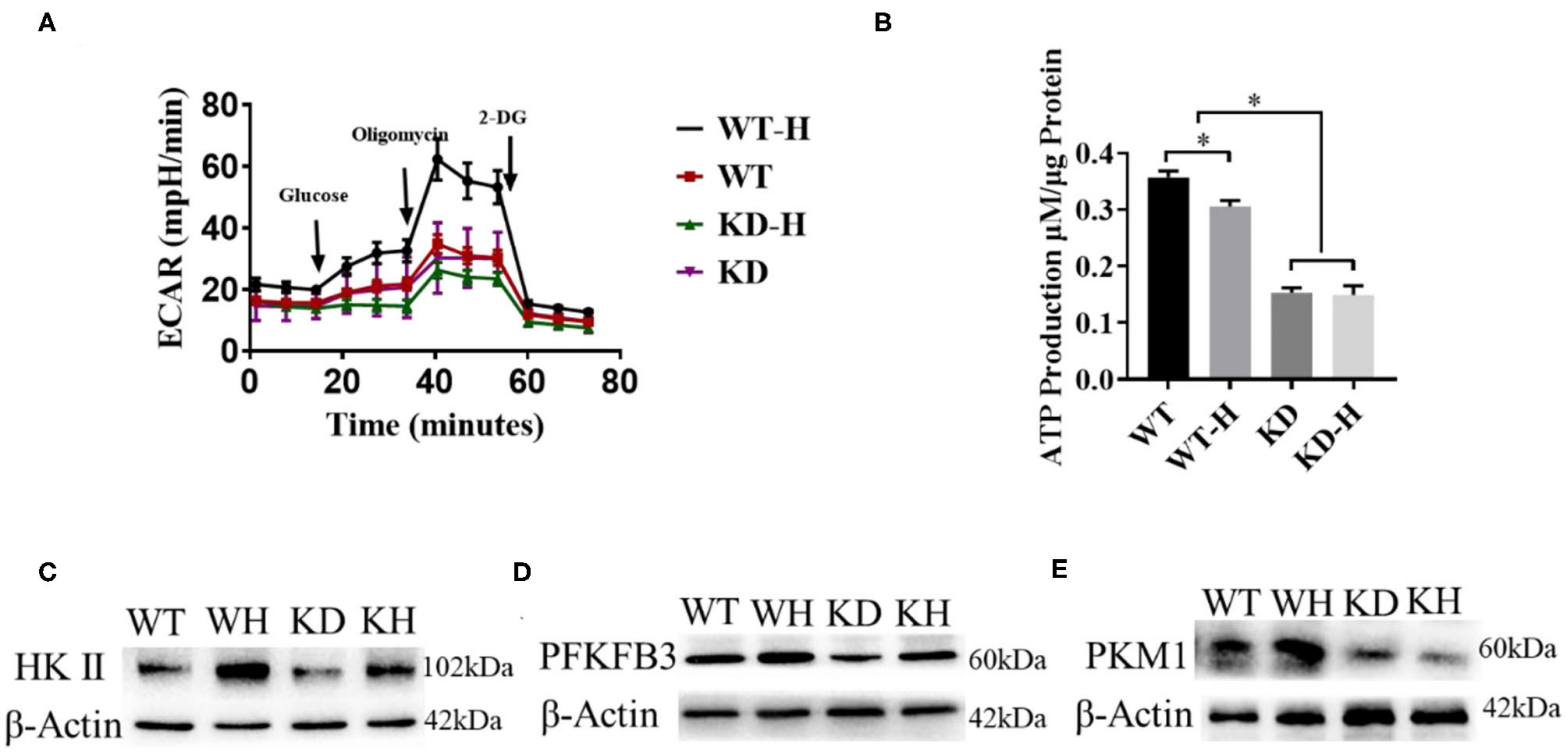
Effect of gamma-aminobutyric acid type B receptor subunit 2 (GABBR2) knockdown on the glycolysis capacity of human umbilical vein endothelial cells (HUVECs) after hypoxia treatment. **(A)** Extracellular acidification rate (ECAR) changes induced by 6 h of hypoxic treatment in normal and GABBR2-downregulated HUVECs. *WT*, normal mice; *KD*, GABBR2-downregulated mice; *WT-H*, wild-type (WT) mice with hindlimb ischemia; *KD-H*, GABBR2 knockdown (KD) mice with hindlimb ischemia. **(B)** ATP production changes induced by 6 h of hypoxic treatment in normal and GABBR2-downregulated HUVECs. **(C–E)** Expression changes of HKII, PFKFB3, and PKM1 induced by 6 h of hypoxic treatment in normal and GABBR2-downregulated HUVECs. *p < 0.05.

It was reported that ECs rely on glycolysis to participate in vessel sprouting (3). To analyze the effect of GABBR2 downregulation on cellular energetics, lentivirus-mediated GABBR2 knockdown in HUVECs was used in our study. Since hypoxia promotes glycolysis, we detected the metabolic changes of HUVECs under normoxic and hypoxic conditions by assessing the ECAR. The results showed that hypoxia treatment remarkably increased the ECARs of HUVECs. However, GABBR2 downregulation inhibited the glycolysis levels of HUVECs under hypoxic condition (Figure 5A). Furthermore, total ATP generation was evaluated in HUVECs with different GABBR2 expressions. The results demonstrated that GABBR2 knockdown significantly decreased cellular ATP production (Figure 5B). HKII, PFKFB3, and PKM1 are three key enzymes responsible for glycolysis. Our results indicated that the expressions of these enzymes were enhanced by hypoxia treatment, which were significantly downregulated by GABBR2 downregulation under normoxic or hypoxic condition (Figures 5C–E). The above results indicated that the inhibitory effect of GABBR2 knockdown on glycolysis capacity could be responsible for the damaged angiogenesis post-ischemia.

## Discussion

The present study demonstrated that the upregulation of GABBR2 induced by hypoxia or ischemia plays an important role in the proliferation, migration, and angiogenesis of HUVECs, which were further proven by the compromised blood recovery in GABBR2-downregulated ischemic hindlimb of mice. Besides, the mobilization of CD34^+^/CD133^+^ cells in peripheral blood was inhibited by hindlimb GABBR2 knockdown in mice. Mechanically, GABBR2 downregulation inhibited the glycolysis capacity of HUVECs by downregulating the expression of glycolysis-associated enzymes. Gamma-aminobutyric acid type B receptor subunit 2 was reported to implicate in a number of neuronal disorders, including cognitive impairments, nociception, anxiety, and depression (19–21). In the neuronal pathophysiology of many CNS diseases and disorders, GABBR2 regulates intracellular signaling by G protein-coupled receptors to activate adenylyl cyclase. However, our study indicated that the critical role of GABBR2 in the process of EC angiogenesis might be attributed to the regulation of the glycolysis pathway. Our results provide a new horizon and mechanism for post-ischemic angiogenesis, which could be a potential therapeutic target of metabolism regulation.

Several modes of vessel formation have been identified, including sprouting angiogenesis, vasculogenesis, and arteriogenesis. Vasculogenesis is defined as the process in which angioblasts differentiate into ECs in the developing mammalian embryo. Subsequent sprouting angiogenesis ensures the expansion of the vascular network. Then, pericytes or vascular smooth muscle cells cover ECs to provide stability and control perfusion, which is known as arteriogenesis (22). In other words, angiogenesis refers to the formation of new blood vessels from preexisting vessels, which was intensively explored in our study. Given the complexity of angiogenesis, the pro-angiogenic factors that play a predominant role in angiogenesis, such as VEGF and CD31, were detected in our study. Our results showed that the expressions of VEGF and CD31 were activated by hypoxia, which was consistent with the enhanced angiogenesis in the ischemic hindlimb. However, GABBR2 knockdown inhibited VEGF and CD31 expressions, as well as post-ischemic angiogenesis in the hindlimb. Taken together, we conclude that the blood recovery of the ischemic hindlimb in mice was majorly regulated by the mode of angiogenesis, which was further modulated by GABBR2.

The repair of healthy adult vessels or the expansion of pathological vessels can be aided by the recruitment of bone marrow-derived cells and/or EPCs to the vascular wall (23, 24). Mobilization of EPCs might represent an effective means to augment the resident population of ECs (25). Different cytokines and growth factors stimulate bone marrow-derived EPCs to circulate in adult peripheral blood, participating in angiogenesis. Vascular endothelial growth factor is an important cytokine for EPC mobilization from the bone marrow into the peripheral circulation (26, 27). In our study, an increased expression of VEGF was observed in the ischemic hindlimb and, accordingly, a significantly enhanced EPC mobilization in peripheral blood. Gamma-aminobutyric acid type B receptor subunit 2 knockdown inhibited VEGF expression and EPC mobilization. The evidences from our study indicated that GABBR2 knockdown blunted EPC mobilization by inhibiting VEGF expression.

Despite the 36 mol of ATP from glucose oxidation vs. 2 mol of ATP from aerobic glycolysis, ECs generate ATP mainly from glycolysis. Furthermore, the switch of ECs from quiescence to vessel branching accelerates aerobic glycolysis (5, 28, 29). Moreover, a hypoxic environment in the hindlimb favors the glycolysis pathway, whereas glycolysis-dominant metabolism also makes ECs more hypoxia-resistant. In our study, the stimulation of glycolysis by hypoxia was accompanied by the upregulation of GABBR2, indicating the potential connection between glycolysis and GABBR2. Given the above fact, the regulating mode of GABBR2 in glycolysis warrants further exploration. The study of De Bock and colleagues reported that the pro-angiogenic molecule VEGF stimulates the expression of the glycolysis activator PFKFB3 (5). Intriguingly, our study demonstrated that VEGF production was induced by hypoxia, but inhibited by GABBR2 knockdown. Consistent with this result, the expressions of the glycolysis activators, namely, HKII, PFKFB3, and PKM1, were upregulated by hypoxia, but downregulated by GABBR2 knockdown. In brief, we conclude that GABBR2 regulated the glycolysis pathway by controlling the secretion of pro-angiogenic molecules.

Human umbilical vein endothelial cell metabolism plays an important role in the formation of new vessels. The importance of metabolism could be described as the engine in a car and the angiogenic signals as the driver. Once metabolism is compromised, new vessel formation was inhibited regardless of EC angiogenic signals (2). The division and migration of ECs for the formation of new vessels demand a large amount of energy. Endothelial cells highly rely on glycolysis instead of oxidative metabolism for ATP production, most of which is produced by the conversion of glucose into lactate (5, 29, 30). Our results indicated that the glycolysis of ECs was further stimulated by hypoxia treatment, which was evidenced by the significantly increased ECARs. Combined with the profile of the glycolysis-dominated metabolism, enhanced glycolysis post-hypoxia was demonstrated. This reaction, however, was inhibited by GABBR2 knockdown with or without hypoxia treatment. The compromised glycolysis metabolism was further illustrated by the decreased expressions of the catalyzing enzymes participating in the metabolic pathway. Our present study indicated that GABBR2 might be a potential therapeutic target in modulating EC metabolism and the process of ischemia in PADs.

## Data Availability Statement

The datasets presented in this study can be found in online repositories. The names of the repository/repositories and accession number(s) can be found below: https://www.proteinatlas.org/ENSG00000136928-GABBR2/cell.

## Ethics Statement

The animal study was reviewed and approved by Hangzhou Medicine College.

## Author Contributions

HZha and JL contributed to the study design. HZha and HZho collected the data. JY and YN performed statistical analysis and conducted the literature search. HZha interpreted the data. HZha, HZho, JY, YN, and JL prepared the manuscript. JL helped with fund collection. All authors contributed to the article and approved the submitted version.

## Conflict of Interest

The authors declare that the research was conducted in the absence of any commercial or financial relationships that could be construed as a potential conflict of interest.

## Publisher's Note

All claims expressed in this article are solely those of the authors and do not necessarily represent those of their affiliated organizations, or those of the publisher, the editors and the reviewers. Any product that may be evaluated in this article, or claim that may be made by its manufacturer, is not guaranteed or endorsed by the publisher.
