# Supplementary figures and images for: Endothelial GABBR2 Regulates Post-ischemic Angiogenesis by Inhibiting the Glycolysis Pathway
Source: Front Cardiovasc Med. 2021 Aug 4;8:696578. doi: 10.3389/fcvm.2021.696578 (PMC8371460; doi:10.3389/fcvm.2021.696578)

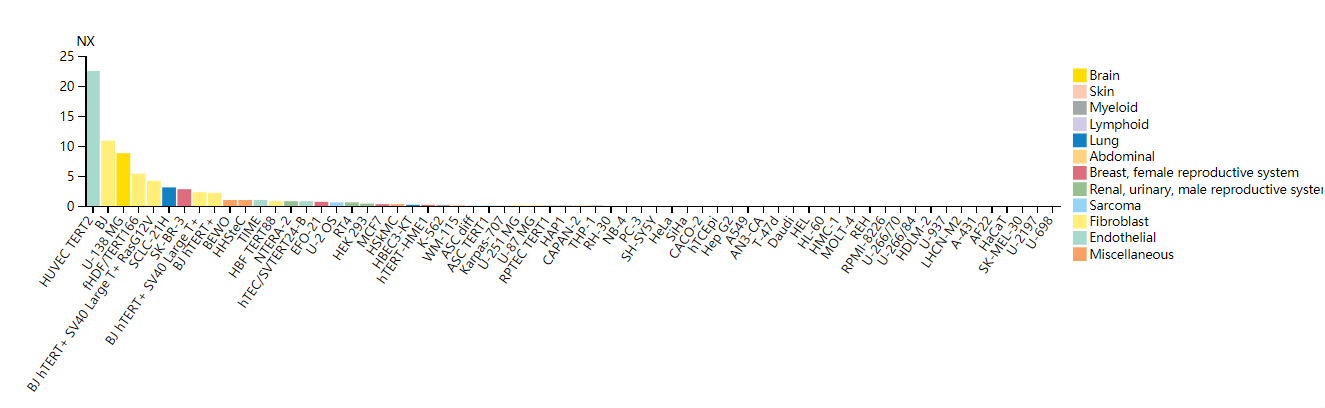

Supplement: Supplementary file 1 [file Data_Sheet_1.ZIP › Figure 1/A RNA-seq data.png]

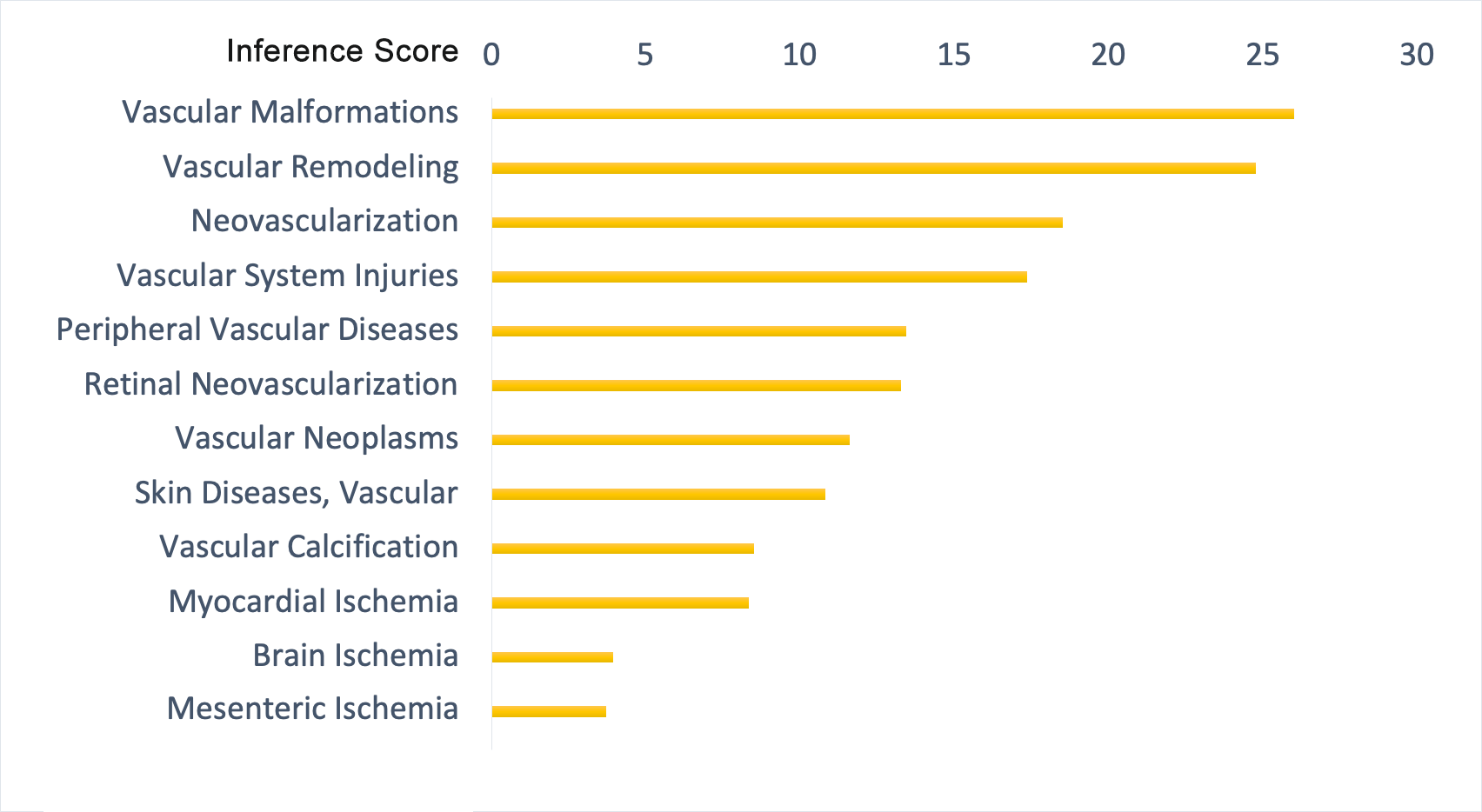

Supplement: Supplementary file 1 [file Data_Sheet_1.ZIP › Figure 1/B Re-speculation the role of GABBR2 in angiogenesis.png]

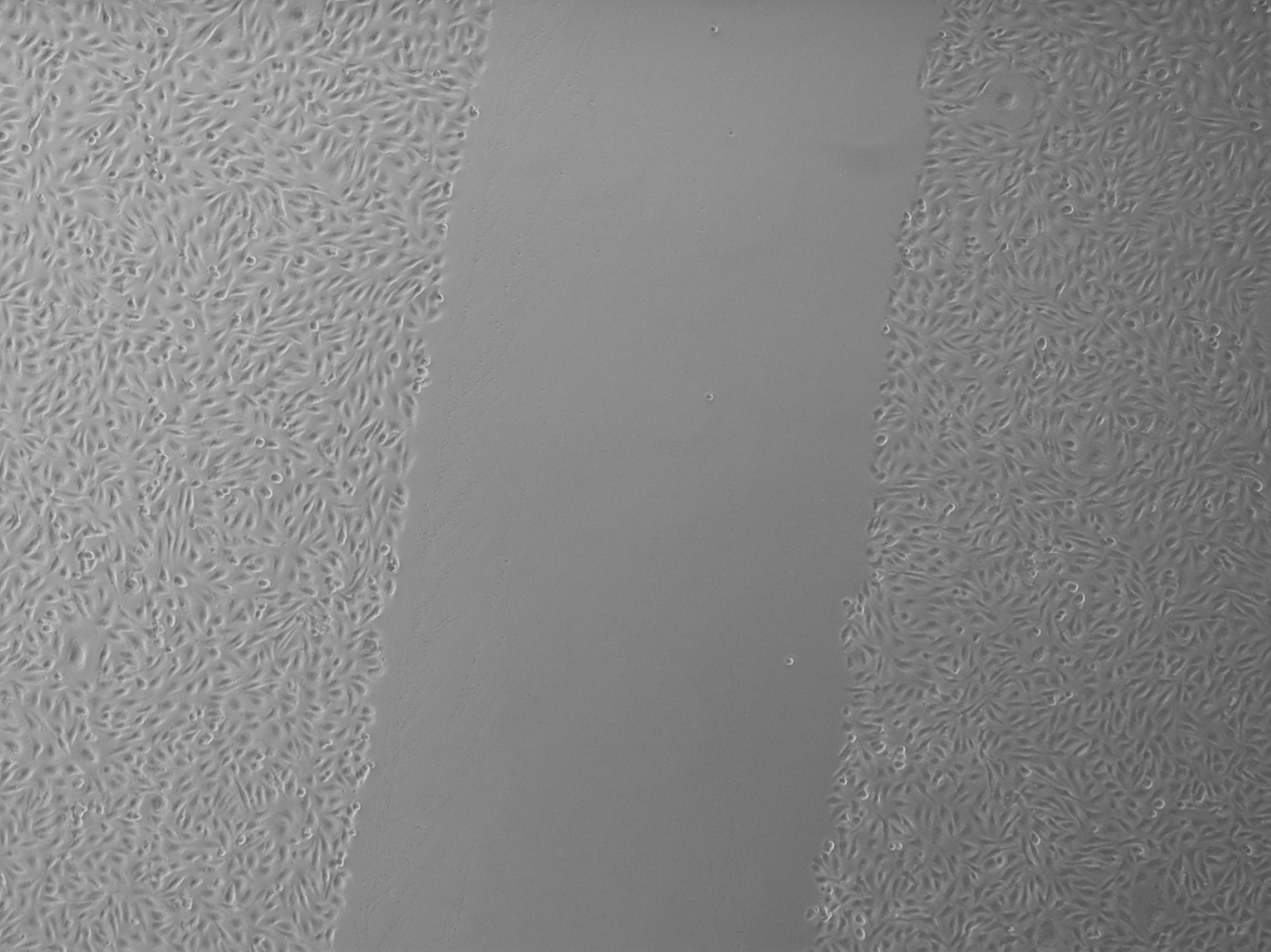

Supplement: Supplementary file 2 [file Data_Sheet_2.ZIP › Figure 2/C/0h/KO-H.jpg]

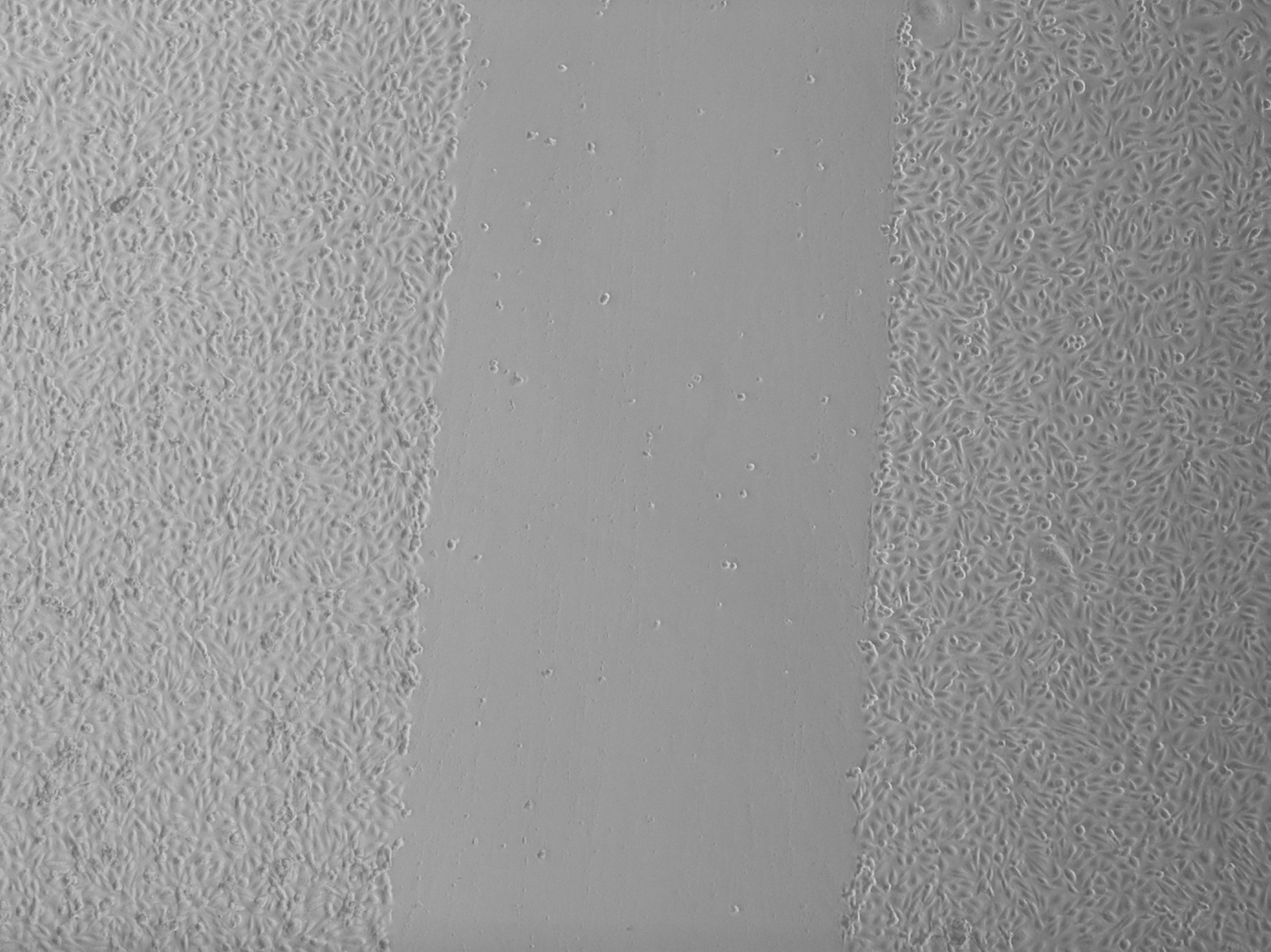

Supplement: Supplementary file 2 [file Data_Sheet_2.ZIP › Figure 2/C/0h/KO.jpg]

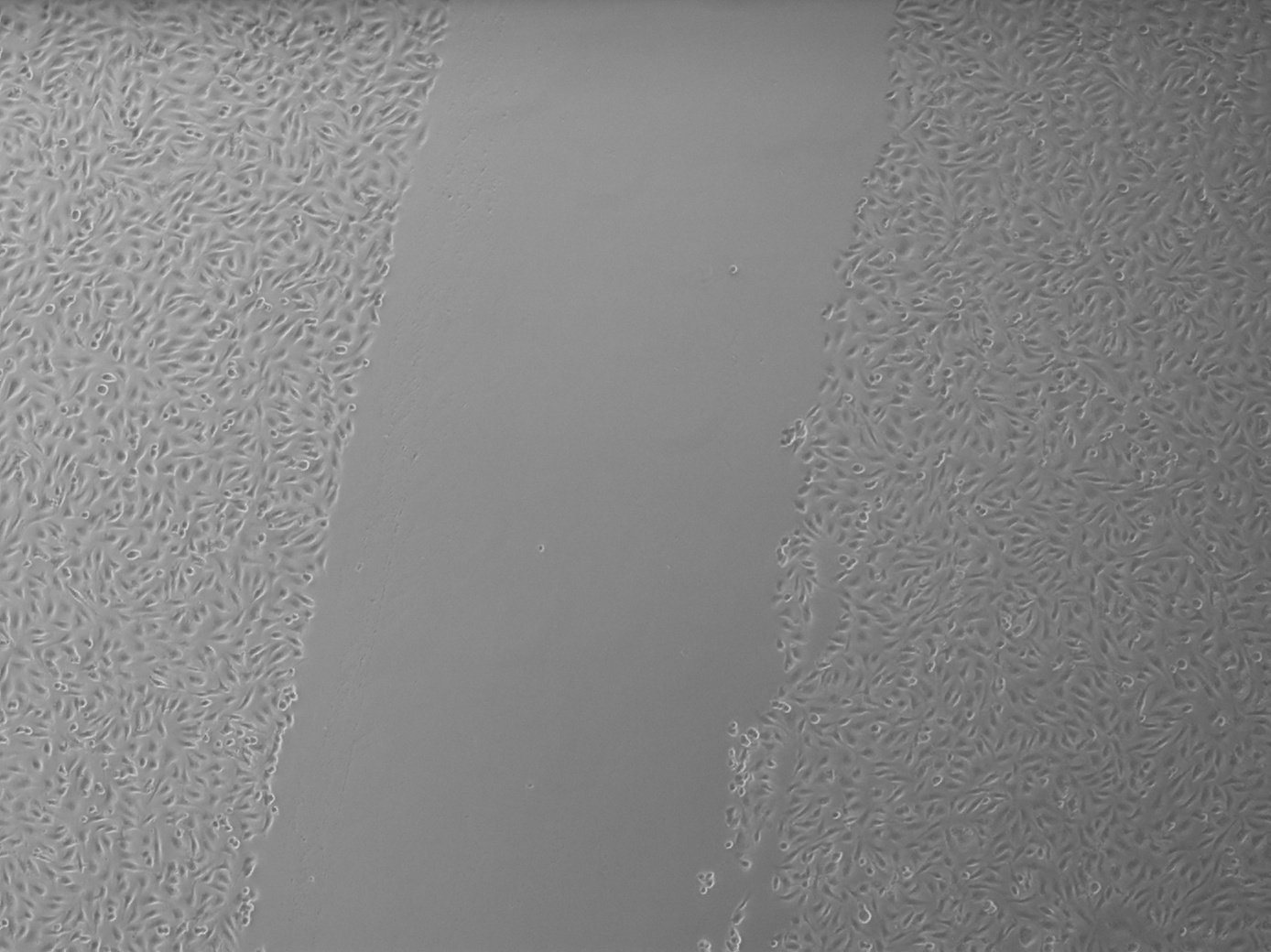

Supplement: Supplementary file 2 [file Data_Sheet_2.ZIP › Figure 2/C/0h/WT-H.jpg]

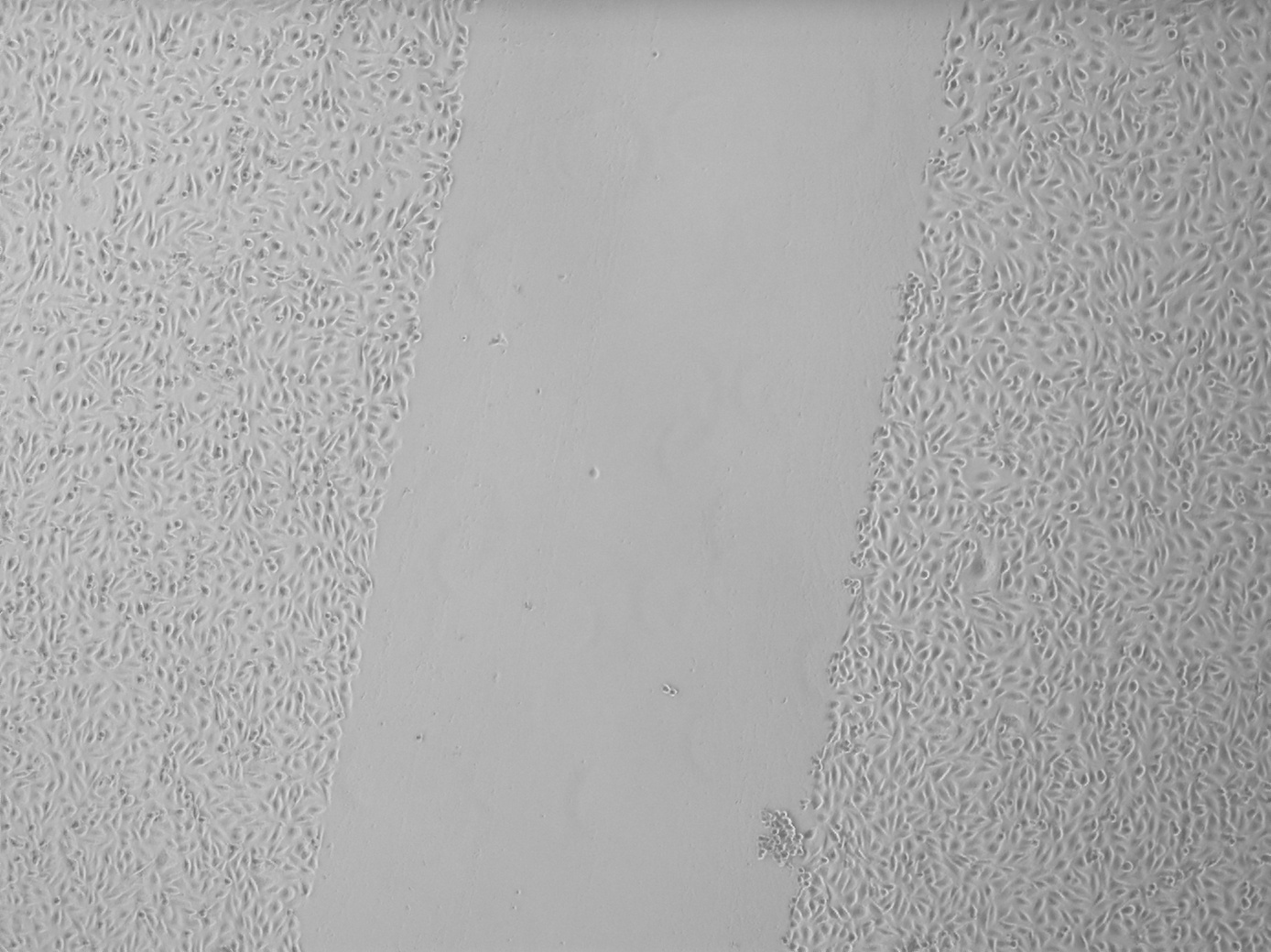

Supplement: Supplementary file 2 [file Data_Sheet_2.ZIP › Figure 2/C/0h/WT.jpg]

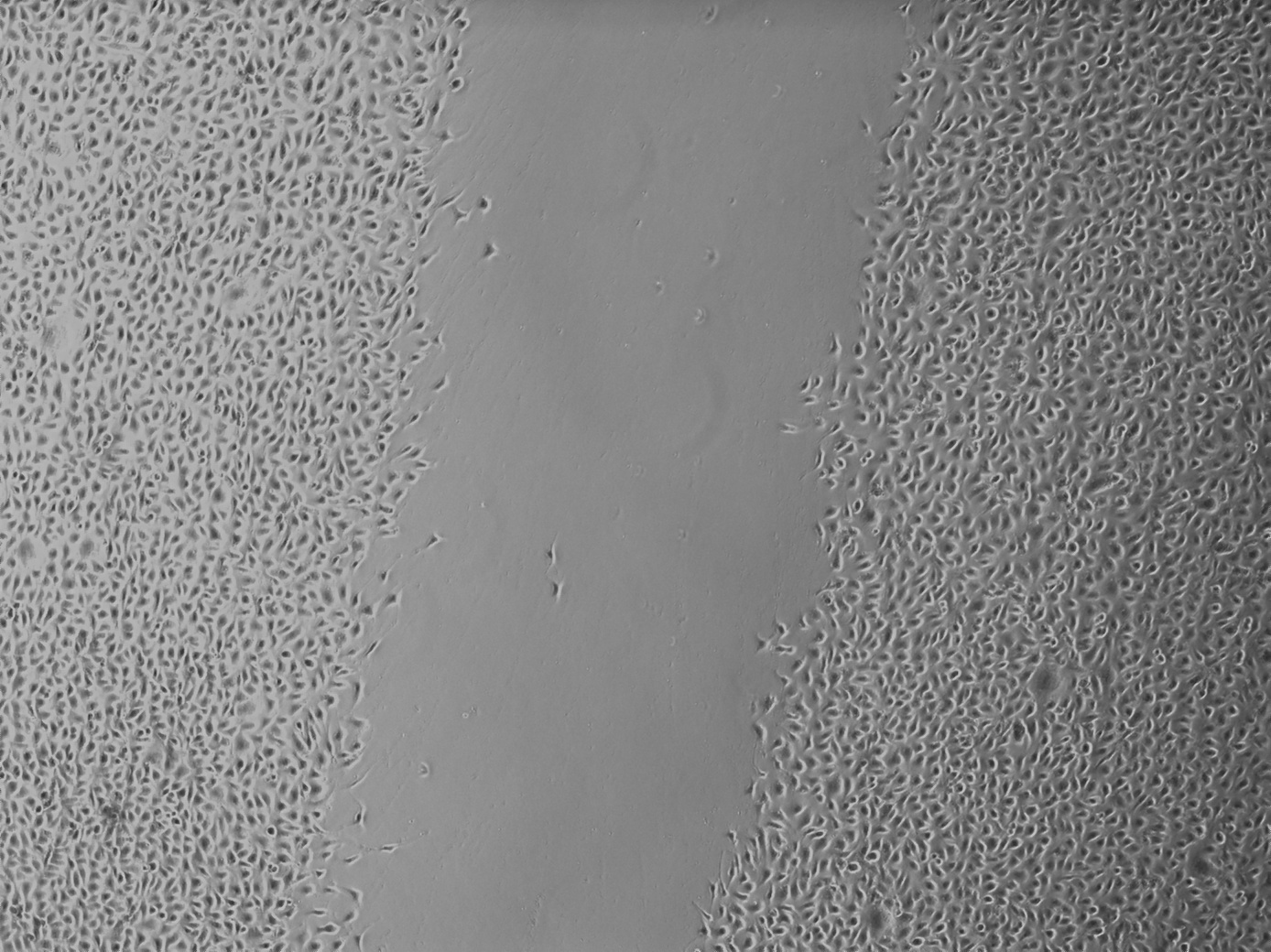

Supplement: Supplementary file 2 [file Data_Sheet_2.ZIP › Figure 2/C/24h/KO-H.jpg]

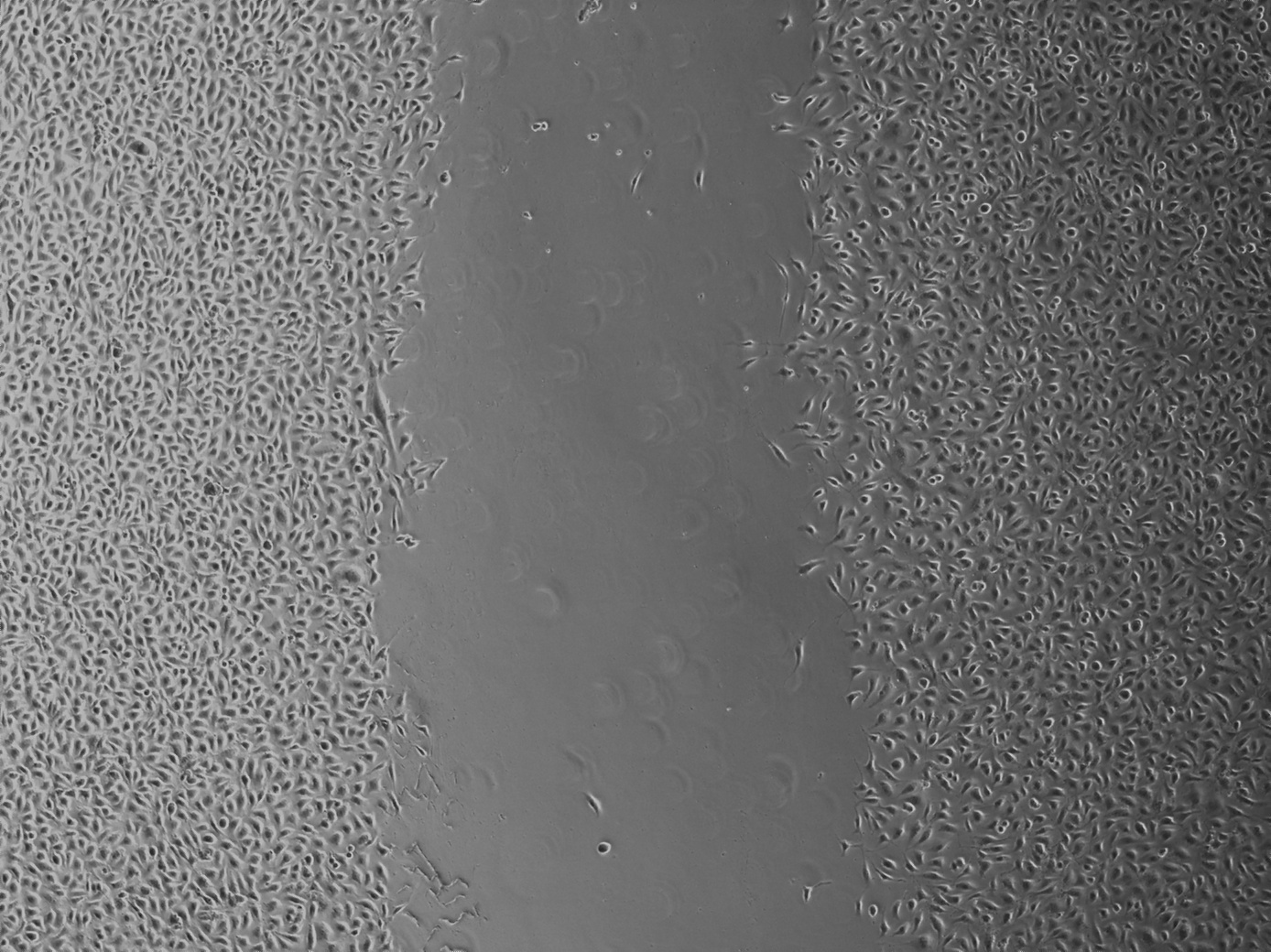

Supplement: Supplementary file 2 [file Data_Sheet_2.ZIP › Figure 2/C/24h/KO.jpg]

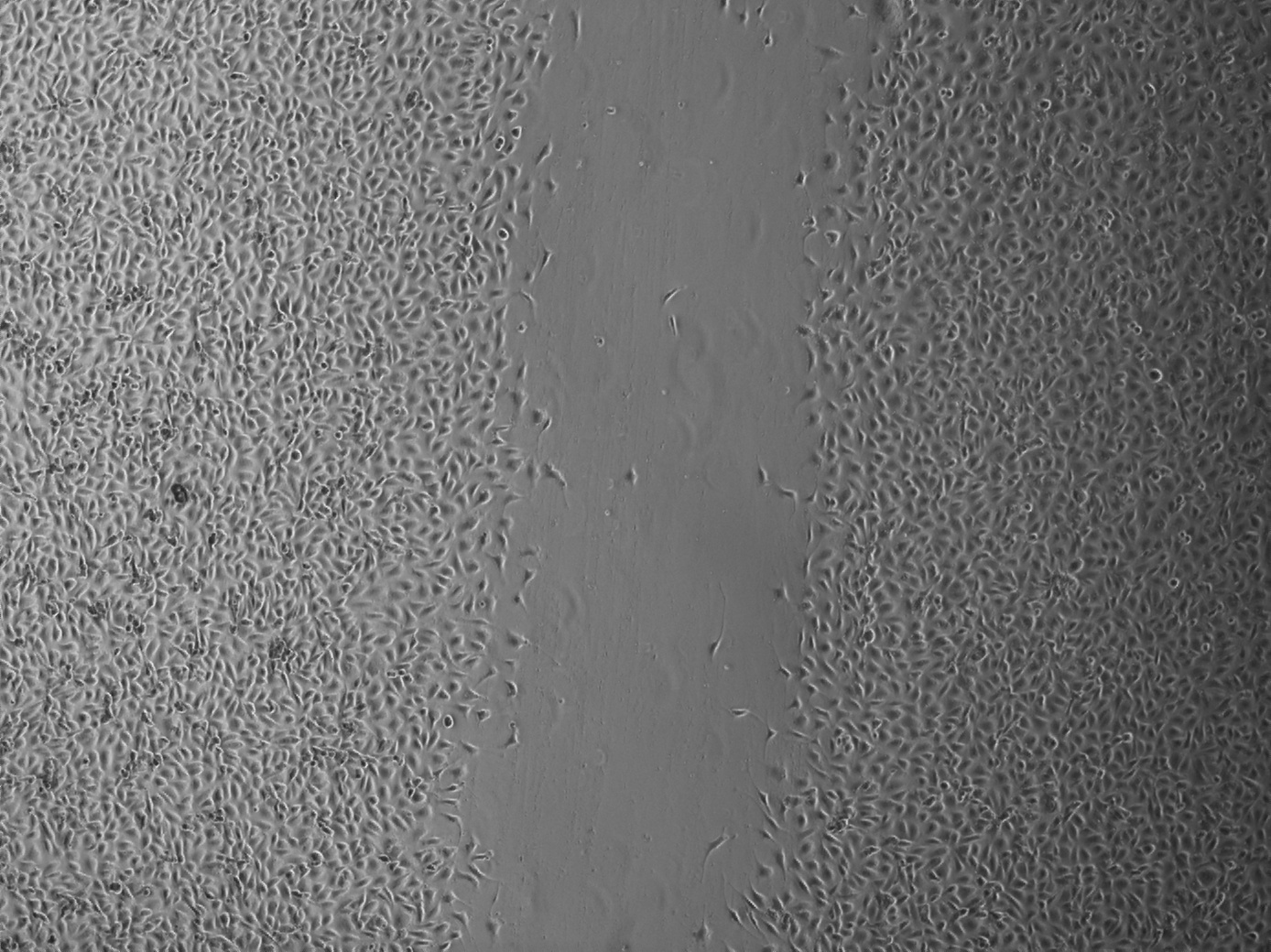

Supplement: Supplementary file 2 [file Data_Sheet_2.ZIP › Figure 2/C/24h/WT-H.jpg]

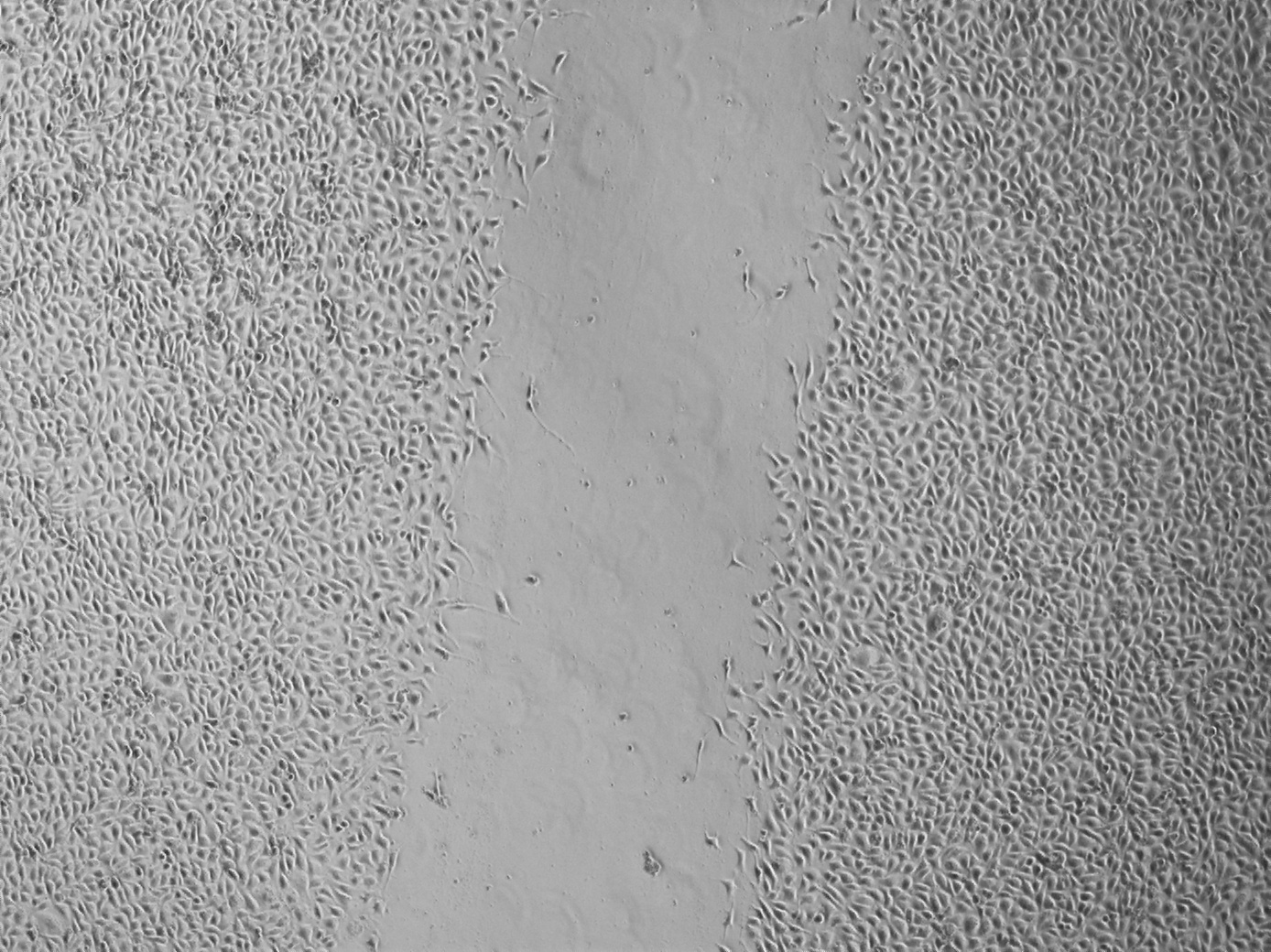

Supplement: Supplementary file 2 [file Data_Sheet_2.ZIP › Figure 2/C/24h/WT.jpg]

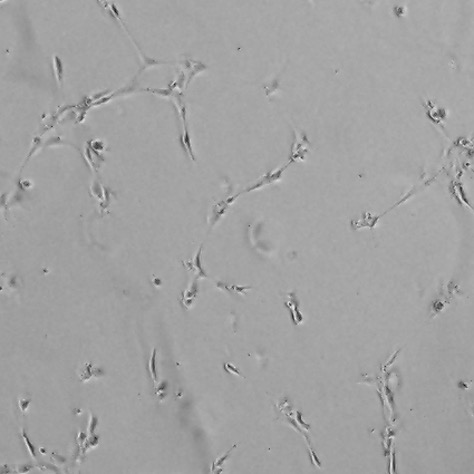

Supplement: Supplementary file 2 [file Data_Sheet_2.ZIP › Figure 2/D/KO-H.jpg]

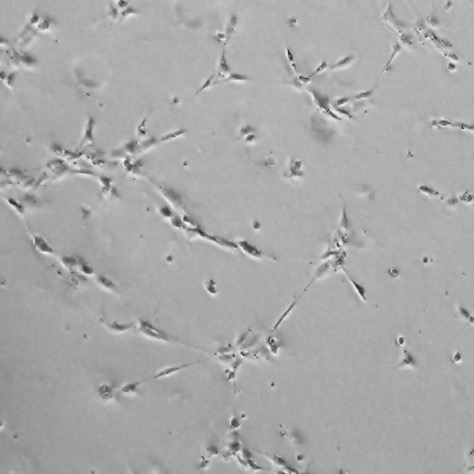

Supplement: Supplementary file 2 [file Data_Sheet_2.ZIP › Figure 2/D/KO.jpg]

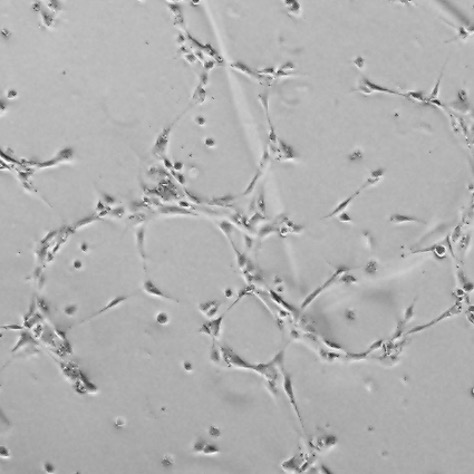

Supplement: Supplementary file 2 [file Data_Sheet_2.ZIP › Figure 2/D/WT-H.jpg]

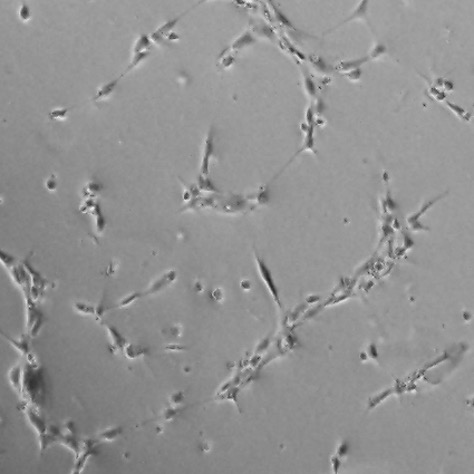

Supplement: Supplementary file 2 [file Data_Sheet_2.ZIP › Figure 2/D/WT.jpg]

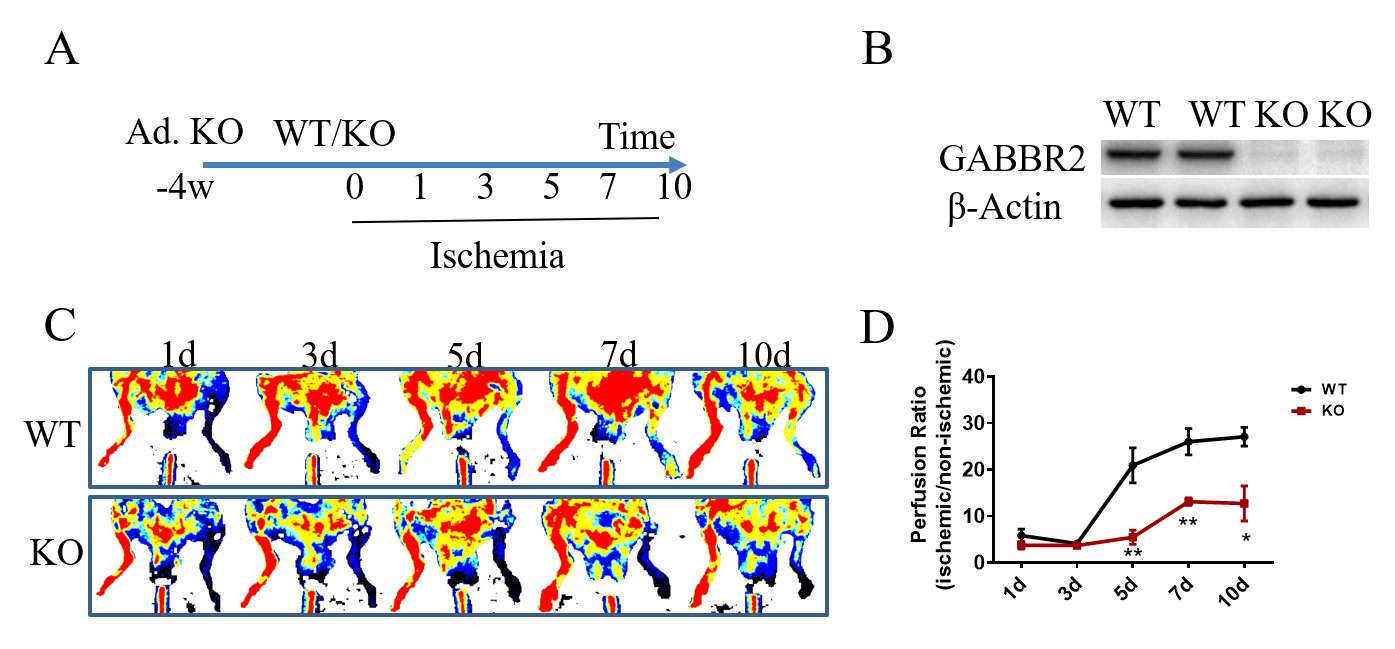

Supplement: Supplementary file 3 [file Data_Sheet_3.ZIP › Figure 3/C/KO/KO-10d.tif]

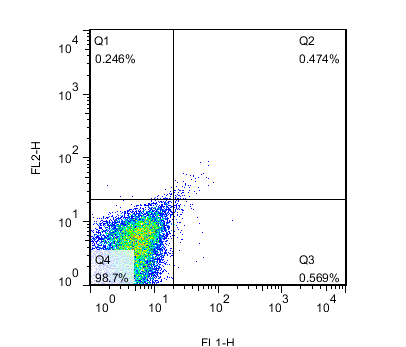

Supplement: Supplementary file 4 [file Data_Sheet_4.ZIP › Figure 4/A/C.png]

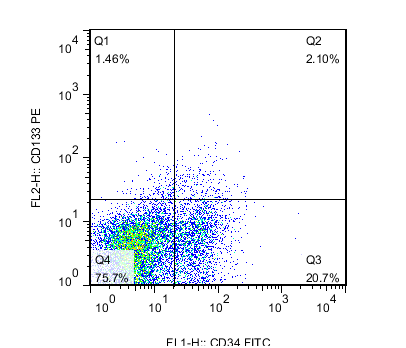

Supplement: Supplementary file 4 [file Data_Sheet_4.ZIP › Figure 4/A/KO-1.png]

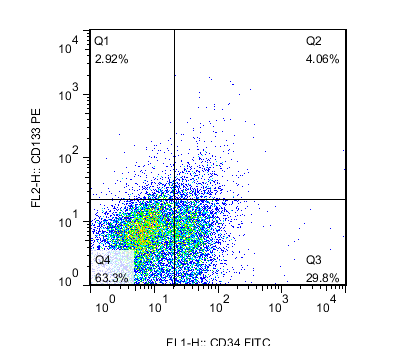

Supplement: Supplementary file 4 [file Data_Sheet_4.ZIP › Figure 4/A/KO-H-1.png]

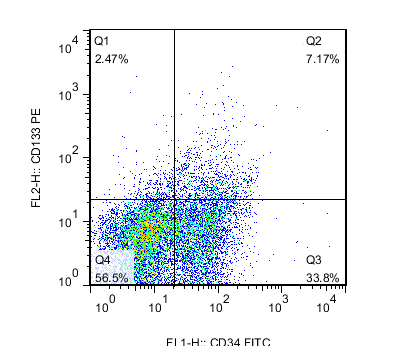

Supplement: Supplementary file 4 [file Data_Sheet_4.ZIP › Figure 4/A/WT-H-1.png]

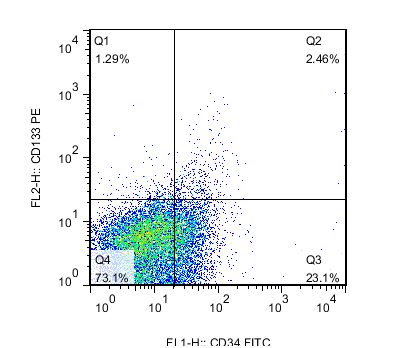

Supplement: Supplementary file 4 [file Data_Sheet_4.ZIP › Figure 4/A/WT1.png]
